# Complete genome of *Listeria aquatica* strain MRL-25-00004 from a cheese ripening environment in Austria

**DOI:** 10.1128/mra.00931-25

**Published:** 2025-10-14

**Authors:** Beatriz Daza Prieto, Johann Ladstaetter, Andrea Murer, Anna Lennkh, Werner Ruppitsch, Ariane Tatjana Pietzka

**Affiliations:** 1 Institute of Medical Microbiology and Hygiene, Austrian Agency for Health and Food Safety 31190, Vienna, Austria; 2 Institute of Food Safety, Austrian Agency for Health and Food Safety 31190, Vienna, Austria; 3 National Reference Laboratory for Listeria, Institute of Medical Microbiology and Hygiene, Austrian Agency for Health and Food Safety 610882 , Graz, Austria; 4 Institute of Hygiene and Medical Microbiology, Medical University Innsbruck 27280, Innsbruck, Austria; 5 Faculty of Food Technology, Food Safety and Ecology, University of Donja Gorica 217861, Podgorica, Montenegro; Wellesley College, Wellesley, Massachusetts, USA

**Keywords:** whole-genome sequencing, cheese, antimicrobial resistance

## Abstract

*Listeria aquatica* was originally isolated from water in Florida (USA) in 2014. Here, we report the complete genome of *L. aquatica* strain MRL-25-00004, which was obtained from lubricating water in a cheese ripening environment in Austria in 2025.

## ANNOUNCEMENT

*Listeria aquatica* was isolated from running water in Florida and described as a novel species in 2014 (1). Currently, eight *L. aquatica* genomes have been deposited on GenBank. Strains were isolated from dairy farms (sheep feces) in Spain (2), cow dung from Bangladesh (3), and soil in the United States (GCF_014223975.1).

In 2025, *L. aquatica* strain MRL-25-00004 was obtained from salt water used to clean cheese wheels from an Austrian cheese ripening facility, following EN ISO 11290 standard (4). Briefly, a 100-mL sample was pre-enriched in half Fraser broth (30°C, 24–26 h), and a 0.1-mL aliquot was used for selective enrichment in the Fraser broth (37°C, 24 h) and plated on ALOA agar (37°C, 24–48 h).

Genomic DNA was extracted from overnight COS cultures (37°C) using the MagAttract HMW DNA Kit (Qiagen, Germany) and the NucleoSpin Microbial DNA Mini Kit (Macherey-Nagel, Germany). Libraries were prepared with the Nextera XT Kit (Illumina, San Diego, CA, USA) and the Rapid Barcoding Kit 24V14 (SQK-RBK114.24) (Oxford Nanopore Technologies, London, UK) and 2 × 300 bp sequenced on a MiSeq as described (5) and on a GridION using a FLO-MIN114 R10.4.1 flow cell, following the manufacturer’s instructions.

A total of 166,880 Nanopore reads (N50: 258,200 bp) were obtained using Dorado v7.1.4 in a superior base-calling mode and filtered using Filtlong v0.2.1 with the following parameters: min_length, 1,000; keep percent, 90; and target_bases, 500,000,000. A total of 1,004,096 Illumina reads were quality controlled using FastQC v0.11.9 (6) and used untrimmed in the final hybrid assembly using Unicycler v0.5 (https://github.com/rrwick/Unicycler), which resulted in 1 contig with a mean coverage of 57-fold, a total length of 2,715,857 bp, and a GC content of 39.43% (Table 1). Unicycler trimmed overlaps at contig-ends and rotated circular replicons using TBLASTN, placing dnaA or repA at the start on the forward strand, ensuring consistent orientation and marking the contig as circular (7).

**TABLE 1:** Characteristics of *L. aquatica* MRL-25-00004

Attribute	Finding
Genome size (Mb)	2.7
No. of contigs	1
No. of short reads	1,004,096
No. of long reads	166,880
Long-read fragment N50 (bp)	2,715,857
GC content (%)	39.3
Genome coverage	57
Total no. of genes	2,718
Pseudo genes	28
Total no. of coding sequences	2,601
No. of RNA genes	89
No. of tRNAs	67
ARGs	*VanY* in *VanA* cluster (33% identity) *VanT* in *VanG* cluster (32% identity)
VGs	*lap* (99% query coverage; 79.95% identity): host cell adhesion *tcsA* (90% query coverage; 83.09% identity): immune modulation *iap* (10% query coverage; 77.42% identity): intracellular survival
*Ad hoc* cgMLST reference genome	Strain FSL S10-1188: GCF_000525795.1
*Ad hoc* cgMLST query genomes	FSL L7-1507: JAARRM000000000 SG_BD1: JBDILX000000000 SM-LAD: JBPOES000000000 CLIP 2021/01678: ERR14212134 CLIP 2020/00417: ERR6496342 CLIP 2019/02937: ERR6496235 CLIP 2019/01686: ERR6496201 MRL-25-00004: CP195758

The NCBI Prokaryotic Genome Annotation v6.6 (8) identified 2,718 genes, 2,601 coding genes, 28 pseudogenes, and 89 RNA genes (Table 1).

MRL-25-00004 was identified as *L. aquatica* using digital DNA-DNA hybridization (dDDH) (formula d4) (https://tygs.dsmz.de/) (9) and average nucleotide identity (10) showing 80.7% (cutoff > 70% dDDH) and 97.45% (cutoff > 95%) identity to *L. aquatica* FSL S10-1188^T^.

Strain comparison with all publicly available *L. aquatica* genomes using an *ad hoc* core genome scheme comprising 1,471 targets (Table 1) showed a minimum of 248 and a maximum of 1,378 allelic differences to CLIP 2021/01678 and FSL S10-1188^T^, respectively.

The Comprehensive Antibiotic Resistance Database (11), tools from the Center for Genomic Epidemiology (https://www.genomicepidemiology.org/, 8 July 2025), and Basic Local Alignment Search Tool v.2.16.0 (12) were used for detecting antimicrobial resistance genes (ARGs), virulence genes (VGs), and plasmids. Prophages were analyzed using PHASTEST v3.0 (13). Default parameters were used unless otherwise specified.

Two ARGs and three VGs were identified in *L. aquatica* MRL-25-00004 (Table 1), and they were associated with host cell adhesion, immune modulation, and intracellular survival. Those VGs have been previously reported in other *L. aquatica* strains (14), suggesting pathogenicity. No plasmids or phages were detected.

## Data Availability

This whole-genome shotgun project has been deposited in the NCBI GenBank database under the accession number CP195758, BioProject accession number PRJNA1288400, and BioSample accession number SAMN49847188. This is the first version of this genome. The raw sequence reads have been deposited in the Sequence Read Archive under the accession numbers SRR34426556 and SRR34428173.
